# Protein Lysine Acetylation in Ovarian Granulosa Cells Affects Metabolic Homeostasis and Clinical Presentations of Women With Polycystic Ovary Syndrome

**DOI:** 10.3389/fcell.2020.567028

**Published:** 2020-09-11

**Authors:** Zheying Min, Xiaoyu Long, Hongcui Zhao, Xiumei Zhen, Rong Li, Mo Li, Yong Fan, Yang Yu, Yue Zhao, Jie Qiao

**Affiliations:** ^1^Center for Reproductive Medicine, Department of Obstetrics and Gynecology, Peking University Third Hospital, Beijing, China; ^2^Peking-Tsinghua Center for Life Sciences, Academy for Advanced Interdisciplinary Studies, Peking University, Beijing, China; ^3^National Clinical Research Center for Obstetrics and Gynecology, Peking University Third Hospital, Beijing, China; ^4^Key Laboratory of Assisted Reproduction, Ministry of Education, Peking University, Beijing, China; ^5^Beijing Key Laboratory of Reproductive Endocrinology and Assisted Reproductive Technology, Beijing, China; ^6^Key Laboratory for Major Obstetric Diseases of Guangdong Province, The Third Affiliated Hospital of Guangzhou Medical University, Guangzhou, China; ^7^Research Units of Comprehensive Diagnosis and Treatment of Oocyte Maturation Arrest, Chinese Academy of Medical Sciences, Beijing, China; ^8^Beijing Advanced Innovation Center for Genomics, Beijing, China

**Keywords:** lysine acetylation, polycystic ovary syndrome, granulosa cells, metabolic pathway, acetyl-CoA acetyltransferase 1

## Abstract

Polycystic ovary syndrome (PCOS) is one of the most common reproductive endocrine disorders accompanied by obvious metabolic abnormalities. Lower-quality oocytes and embryos are often found in PCOS women during assisted reproductive technology treatment. However, there is still no clarity about the mechanism of ovarian metabolic disorders and the impact on oocyte maturation in PCOS. The aim of this study was to understand the potential effect of the posttranslational modification on ovarian metabolic homeostasis and oocyte development potential in women with PCOS. A quantitative analysis of acetylated proteomics in ovarian granulosa cells of PCOS and control groups was carried out by mass spectrometry. There was widespread lysine acetylation of proteins, of which 265 proteins had increased levels of acetylation and 68 proteins had decreased levels of acetylation in the PCOS group. Most notably, differentially acetylated proteins were significantly enriched in the metabolic pathways of glycolysis, fatty acid degradation, TCA cycle, tryptophan metabolism, and branched-chain amino acid degradation. Acetyl-CoA acetyltransferase 1 (ACAT1) was an enzyme central to these metabolic pathways with increased acetylation level in the PCOS group, and there was a negative correlation of ACAT1 acetylation levels in PCOS granulosa cells with oocyte quality and embryo development efficiency in the clinic. Lysine acetylation changes of key enzymes in PCOS granulosa cells might attenuate their activities and alter metabolic homeostasis of follicular microenvironment for oocyte maturation and embryo development.

## Introduction

Polycystic ovary syndrome (PCOS) is a complex and heterogeneous endocrine disorder. Its prevalence is approximately 5%∼10% in reproductive-aged women, and PCOS is the leading cause of anovulatory infertility (Norman et al., 2007; Goodarzi et al., 2011). Except for the reproductive abnormalities, women with this syndrome are often accompanied by metabolic disorders. Obesity, insulin resistance, dyslipidemia, and metabolic syndrome have been recognized as the risk factors for diabetes mellitus and cardiovascular disease in PCOS (Guo et al., 2010; Moran et al., 2010; Tziomalos and Dinas, 2018). These systemic metabolic disorders affect the microenvironmental homeostasis in the ovary and therefore interfere with follicular development and oocyte maturation. The clinical pregnancy rate for PCOS patients is not comparable to that of non-PCOS patients due to the lower quality of oocytes, even though more oocytes are retrieved from women with PCOS in assisted reproductive technology (Qiao and Feng, 2011). Importantly, since women with PCOS often have heterogeneous characteristics, it is difficult to predict their oocyte and embryo developmental outcomes using only one or more genes or to make effective improvements (Louwers et al., 2013). Thus, it is necessary to further understand the differences of the ovarian microenvironment between PCOS and non-PCOS women and to elucidate the potential regulatory mechanism from a macroscopic perspective.

Some previous researches have screened the differentially expressed genes and non-coding RNAs in the granulosa cells to explore the regulatory mechanism of PCOS development, and these key differential genes were mainly enriched in the pathways related to metabolism, steroidogenesis, inflammation, cell proliferation, and apoptosis (Abedel-Majed et al., 2019; Li et al., 2019; Che et al., 2020). In addition to the transcriptional regulation, posttranslational modifications (PTMs) are covalent processing events that are catalyzed by enzymes after protein translation by ribosomes is complete. PTMs play a vital role in altering the protein activity state, localization, turnover, and interactions with other proteins; thus, PTMs are crucial mechanisms for regulating protein properties and increasing the functional diversity of the proteome (Stram and Payne, 2016). Lysine acetylation is a conserved PTM that links acetyl-coenzyme A (acetyl-CoA) metabolism to cellular signaling. During the past decade, developments in proteomic technologies and tools have strongly contributed to the rapid progress of acetylation research (Choudhary et al., 2014). Many proteomic analyses have identified thousands of lysine acetylation sites distributed over 1500 proteins (Hernandez-Saavedra et al., 2019; Wang et al., 2019). Lysine residues are often present in the active sites of enzymes, and their acetylation/deacetylation has been recognized to be involved in regulating the catalytic activity of the types of enzymes involved in cellular metabolism (Hernandez-Saavedra et al., 2019; Wang et al., 2019), including glycolysis, gluconeogenesis, fatty acid oxidation, and the tricarboxylic acid (TCA) cycle. Therefore, protein acetylation plays an important role in the occurrence and development of metabolic-related diseases, such as diabetes, cardiovascular disease, metabolic syndrome, and tumorigenesis (Lv et al., 2011; Lin et al., 2013; Lee et al., 2014).

In recent years, our previous metabolomics studies have shown abnormal levels of metabolites in both the sera and follicular fluids of PCOS patients, and these metabolites are involved in various important metabolic pathways (Zhao et al., 2012; Zhao H. et al., 2015; Min et al., 2018). However, the regulatory mechanisms of these metabolic abnormalities and their effects on reproductive function have not yet been clearly elucidated. Based on this, the purpose of this study was to comparatively profile protein lysine acetylation in ovarian granulosa cells from PCOS women and to investigate and understand the mechanism of local metabolic disorder in the ovary and its effect on oocyte development. Our data demonstrated that there was widespread protein lysine acetylation in the granulosa cells of PCOS women and that lysine acetylation mainly regulated several enzymes involved in glycolysis, fatty acid degradation, TCA cycle, tryptophan metabolism, and branched-chain amino acid degradation. Lysine acetylation changes of key enzymes in PCOS granulosa cells attenuated their activities and altered metabolic homeostasis of the follicular microenvironment for oocyte maturation, which provided a new and important mechanism that regulated the ovarian metabolic disorders in PCOS.

## Materials and Methods

### Subjects

The subjects in this study consisted of PCOS and healthy women who visited the Reproductive Medicine Center of Peking University Third Hospital from April to November 2017. PCOS was diagnosed according to the 2003 Rotterdam Criteria, i.e., the presence of two or more of oligo-ovulation and/or anovulation cycles, clinical and/or biochemical signs of hyperandrogenism, and polycystic ovaries after exclusion of other etiologies. The control subjects were selected from women attending the clinic on account of male azoospermia. All of the controls had regular menstrual cycles and normal ovarian morphology, and none exhibited clinical or biochemical hyperandrogenism. All subjects were free of medications known to affect metabolic function or reproductive function within 3 months preceding enrollment. This study was approved by the Reproductive Medicine Ethics Committee of Peking University Third Hospital (2016SZ-027). Informed consent after a detailed explanation of the procedure was acquired from each participant.

### Isolation of Ovarian Mural Granulosa Cells

Patients underwent *in vitro* fertilization with the same stimulation protocol as previously described (Hu et al., 2019). Mural granulosa cells from PCOS and control subjects were isolated from the follicular fluid aspirated during oocyte retrieval by Ficoll density gradient centrifugation (Zhao Y. et al., 2015), and then the cells were lysed directly for protein extraction and analysis.

### Protein Extraction, Trypsin Digestion, and Acetyl Peptide Enrichment

The granulosa cells were collected from 47 PCOS patients and 55 controls. The samples of two groups were mixed separately, and quantitative acetylated proteomics analysis was performed. There were three technical replicates. The sample was sonicated three times on ice using a high-intensity ultrasonic processor (Scientz) in lysis buffer (8 M urea, 1% Protease Inhibitor Cocktail) (Note: For PTM experiments, inhibitors were also added to the lysis buffer, e.g., 3 μM TSA and 50 mM NAM for acetylation). The remaining debris was removed by centrifugation at 12,000 g at 4°C for 10 min. Finally, the supernatant was collected, and the protein concentration was determined with a BCA kit according to the manufacturer’s instructions.

For digestion, the protein solution was reduced with 5 mM dithiothreitol for 30 min at 56°C and alkylated with 11 mM iodoacetamide for 15 min at room temperature in darkness. The protein sample was then diluted by adding 100 mM NH_4_HCO_3_ to urea concentration less than 2 M. Finally, trypsin was added at a 1:50 trypsin-to-protein mass ratio for the first digestion overnight and a 1:100 trypsin-to-protein mass ratio for a second 4-h digestion.

To enrich lysine acetylation-modified peptides, tryptic peptides dissolved in NETN buffer (100 mM NaCl, 1 mM EDTA, 50 mM Tris–HCl, 0.5% NP-40, pH 8.0) were incubated with prewashed antibody beads (PTM-101, PTM Bio) at 4°C overnight with gentle shaking. Then, the beads were washed four times with NETN buffer and twice with H_2_O. The bound peptides were eluted from the beads with 0.1% trifluoroacetic acid. Finally, the eluted fractions were combined and vacuum-dried. For LC-MS/MS analysis, the resulting peptides were desalted with C18 ZipTips (Millipore) according to the manufacturer’s instructions.

### Mass Spectrometry-Based Global Analysis of Lysine Acetylation

The tryptic peptides were dissolved in 0.1% formic acid (solvent A) and directly loaded onto a homemade reversed-phase analytical column (15-cm length, 75 μm i.d.). The gradient was comprised of a solvent B (0.1% formic acid in 98% acetonitrile) increase from 6% to 23% over 26 min, an increase from 23% to 35% over 8 min, a climb to 80% over 3 min, and a hold at 80% for the last 3 min, all at a constant flow rate of 400 nL/min on an EASY-nLC 1000 UPLC system.

The peptides were subjected to an NSI source followed by tandem mass spectrometry (MS/MS) in Q Exactive^TM^ Plus (Thermo) coupled online to the UPLC. The electrospray voltage applied was 2.0 kV. The m/z scan range was 350 to 1800 for a full scan, and intact peptides were detected in the Orbitrap at a resolution of 70,000. Peptides were then selected for MS/MS using the NCE setting as 28, and the fragments were detected in the Orbitrap at a resolution of 17,500. A data-dependent procedure that alternated between one MS scan followed by 20 MS/MS scans with a 15.0-s dynamic exclusion was used. The automatic gain control (AGC) was set at 5E4.

The labile-free method was used to quantify the acetyl proteomics. The raw mass spectrometric data had been deposited and could be accessed using the following URL: http://msviewer.ucsf.edu/prospector/cgi-bin/msform.cgi?form=msviewer (the search key for the saved data set is q40sd3k4bg).

### Immunoprecipitation and Western Blotting

The ovarian granulosa cells were lysed with TNE buffer (50 mM Tris–HCl, pH 7.5, 100 mM NaCl, 1 mM EDTA, and 0.5% NP-40). One microgram of antibody was added and incubated at 4°C for 12 h with gentle shaking. The protein A magnetic beads were added and incubated at 4°C for 1 h. Then, the beads were washed with TNE buffer five times and boiled at 100°C for 5 min. The granulosa cell samples of 3 PCOS patients and 3 controls were tested by western blotting with an anti-acetylated antibody (PTM-105, PTM Biolabs) to verify the changes of protein lysine acetylation, and the granulosa cell samples of 25 PCOS patients were examined to analyze the correlation of the lysine acetylation of ACAT1 protein to the clinical outcomes. Antibodies included PGK1 (17811-1-AP, Proteintech), PGAM1 (ab129191, Abcam), GAPDH (ab181602, Abcam), and ACAT1 (#44276, Cell Signaling Technology).

### Bioinformatics Analysis

The proteomic data were searched by MaxQuant database (v.1.5.2.8). The parameters were SwissProt Human (20130 sequences). We used a subcellular localization predication software, WoLF PSORT, to predict subcellular localization. The STRING database system was used to construct a protein–protein interaction network. Cluster membership was visualized by a heat map using the “heatmap.2” function from the “gplots” R-package. The Gene Ontology (GO) annotation proteome was derived from the UniProt-GOA database (http://www.ebi.ac.uk/GOA/). The Kyoto Encyclopedia of Genes and Genomes (KEGG) database was used to annotate the protein pathways. A corrected *p*-value < 0.05 was considered significant. In motif analysis, Soft motif-x was used to analyze the model of sequences constituted with amino acids in specific positions of modify-21-mers (10 amino acids upstream and downstream of the site) in all protein sequences. Correlation analysis of acetylation level and clinical results was based on Pearson correlation analysis. ImageJ was used to analyze the grayscale of Western blotting.

## Results

### Baseline Characteristics of the PCOS and Control Subjects

We collected the ovarian granulosa cells from 47 PCOS patients and 55 non-PCOS controls. The baseline characteristics of the PCOS and control groups are described in Table 1. These two groups had similar ages and BMIs. The women in the PCOS group had much longer menstrual cycles than those in the control group. In addition, there was a broad spectrum of endocrine and metabolic changes in the PCOS group compared with the control group, including obviously increased triglyceride and low-density lipoprotein (LDL) levels, reduced high-density lipoprotein (HDL) levels, marked increases in the serum concentrations of androgens including testosterone and androstenedione, and an increased ratio of luteinizing hormone (LH) to follicle-stimulating hormone (FSH). These changes were consistent with the clinical phenotypes of PCOS.

**TABLE 1:** Clinical parameter in women with PCOS and control groups.

**Parameters**	**PCOS (47)**	**Control (55)**	***p*-value**
Age at surgery (years)	29.4 ± 3.9	30.0 ± 3.5	ns
BMI (kg/m^2^)	23.4 ± 3.1	21.7 ± 3.7	ns
Menstrual cycle (days)	60.2 ± 40.9	29.5 ± 2.2	<0.01
FSH (mIU/ml)	5.6 ± 1.9	6.6 ± 1.8	<0.01
LH(mIU/ml)	9.6 ± 6.5	4.0 ± 2.0	<0.01
LH/FSH	1.6 ± 1.1	0.6 ± 0.4	<0.01
Estradiol (pmol/L)	230.6 ± 89.8	166.6 ± 89.7	<0.01
Progesterone (nmol/L)	3.0 ± 3.7	1.2 ± 0.4	ns
Testosterone (nmol/L)	1.3 ± 1.0	0.7 ± 0.2	<0.01
Androstenedione (nmol/L)	11.1 ± 4.4	6.6 ± 3.5	<0.01
Fasting Glucose (mmol/L)	4.9 ± 0.5	5.2 ± 0.4	ns
Fasting Insulin (mU/L)	13.5 ± 10.0	—	—
HOMA-IR	3.0 ± 2.2	—	—
Total cholesterol (mmol/L)	4.8 ± 0.87	4.2 ± 0.92	<0.01
Triglyceride (mmol/L)	1.5 ± 1.0	0.9 ± 0.5	<0.01
HDL-C (mmol/L)	1.3 ± 0.2	1.4 ± 0.3	0.05
LDL-C (mmol/L)	3.1 ± 0.8	2.6 ± 0.6	<0.01

*p-values > 0.05 were considered non-significant (ns). LH, luteinizing hormone; FSH, follicle-stimulating hormone; LDL, low-density lipoprotein; HDL, high-density lipoprotein.*

### Profiling of Lysine Acetylated Proteins in Ovarian Granulosa Cells

**FIGURE 1:**
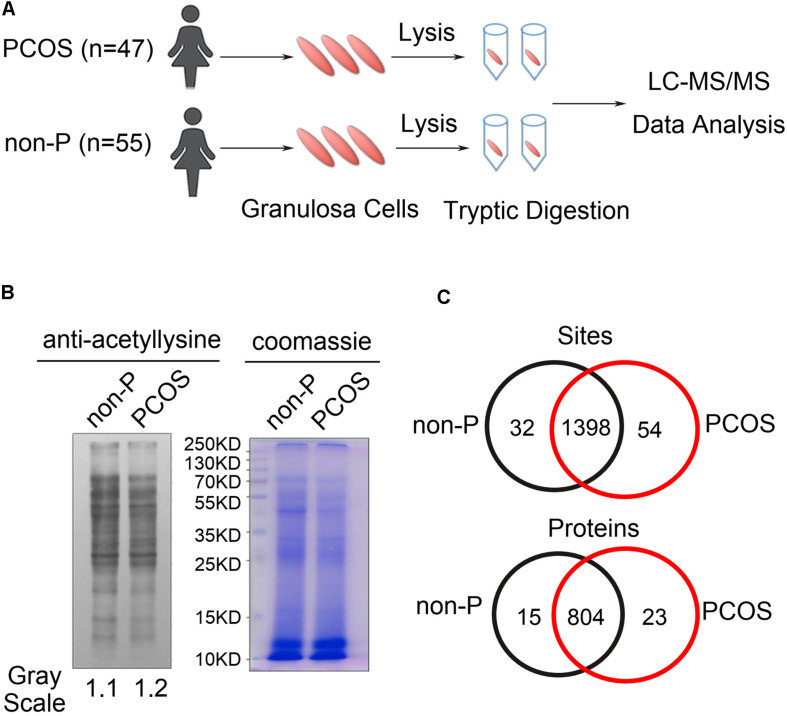
Detection of lysine acetylation in granulosa cells of PCOS and control groups. **(A)** Diagram of granulosa cell collection, lysis, and LC-MS/MS analysis. **(B)** Western blot analysis of granulosa cell lysates from PCOS (*n* = 47) and control (*n* = 55) women by anti-acetyl lysine antibody. Granulosa cells in each group were combined and examined. The loading was controlled by Coomassie blue staining. **(C)** Venn diagram showed overlapped acetylated sites and proteins between PCOS and control groups.

To comprehensively detect the changes in protein lysine acetylation in ovarian granulosa cells, a quantitative acetylated proteomics analysis was carried out according to the flowchart shown in Figure 1A. The granulosa cell samples in 47 PCOS patients and 55 non-PCOS controls were mixed separately. We first measured the lysine acetylation levels in the total protein sample of the two groups and found widespread protein lysine acetylation in the granulosa cells (Figure 1B). The quantitative profile of protein lysine acetylation by LC-MS/MS was performed in triplicate granulosa cells from the PCOS and control groups. The results showed that there were 1398 acetylated sites and 804 acetylated proteins overlapped between the two groups (Figure 1C). Pearson correlation analysis indicated that three technical parallel replicates had a good repeatability (Supplementary Figure 1), and the length of peptides agreed with the quality of tryptic peptides (Supplementary Figure 2).

### Identification of Differentially Acetylated Proteins in PCOS Granulosa Cells

**FIGURE 2:**
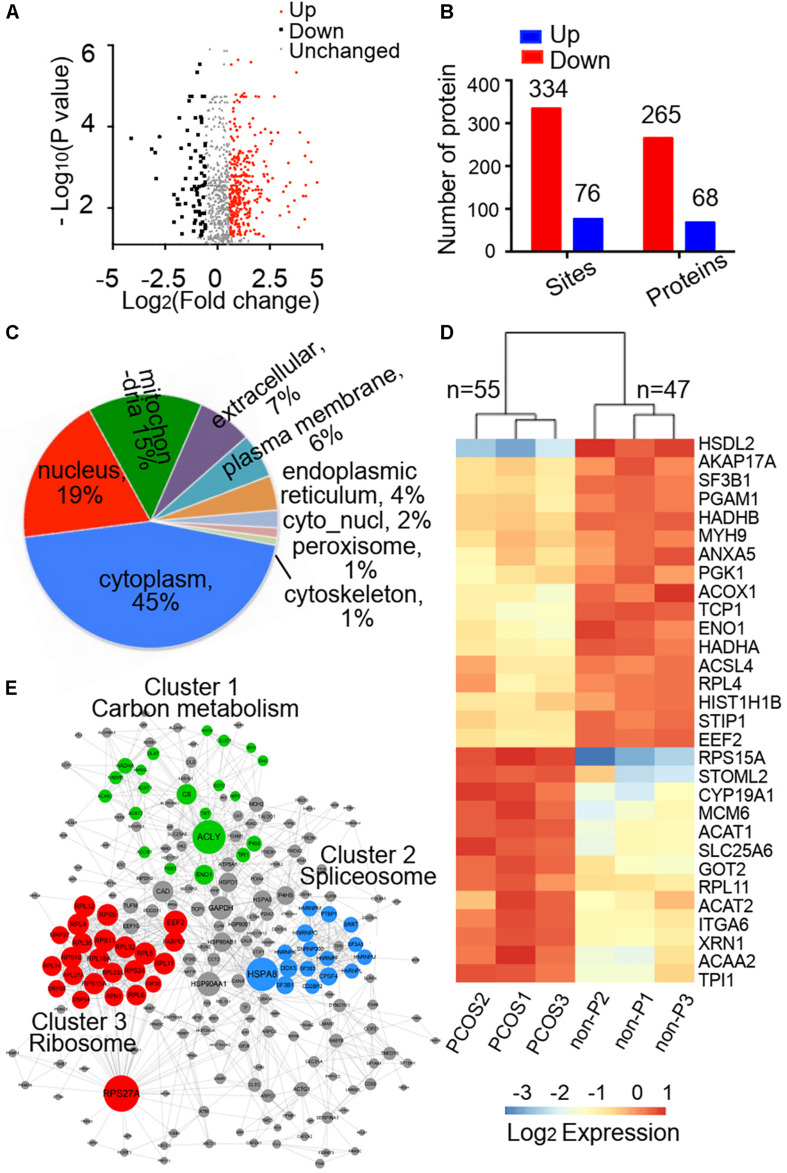
Identification of differentially acetylated proteins and bioinformatics analysis. **(A)** Volcano plot showing differential acetylated proteins between PCOS and control groups, and the differential expression multiples were more than 1.5. **(B)** Number of differentially acetylated sites and proteins in the PCOS group when compared with the control group. **(C)** Subcellular localization predication of differentially acetylated proteins using WoLF PSORT software. **(D)** Heatmap of top 30 of differentially acetylated proteins. The clustering results were shown in triplicate samples from PCOS and control groups. **(E)** Protein–protein interaction network analysis of differentially acetylated proteins extracted from the STRING database.

In total, 410 sites on 333 proteins were identified differentially acetylated when the differential expression multiples were more than 1.5 between PCOS and control groups, of which 334 sites on 265 proteins had increased levels of acetylation and 76 sites on 68 proteins had decreased levels of acetylation in PCOS group (Figures 2A,B and Supplementary Table 1). The subcellular locations of these differentially acetylated proteins were extensively distributed in the cytoplasm, nucleus, and mitochondria (Figure 2C). The characteristics of specific acetylated peptides were evaluated by motif analysis (Supplementary Figure 3). Phenylalanine (F), histidine (H), serine (S), and asparagine (N) were prone to appear in the positions flanking the lysine acetylation sites. Moreover, the quantitative analysis of the top differentially acetylated proteins was shown in triplicate granulosa cells from PCOS and control groups. The clustering results revealed that proteins with significant differences in acetylation levels in the PCOS group consisted of many enzymes in metabolic regulation (Figure 2D). To better understand the function of lysine acetylation, the differentially acetylated proteins were analyzed using a protein–protein interaction network. Three highly connected clusters were identified, including carbon metabolism, ribosome, and spliceosome (Figure 2E), which indicated that lysine acetylation occurred in various metabolic complexes.

### Functional Annotation and Enrichment Analysis of Differentially Acetylated Proteins

**FIGURE 3:**
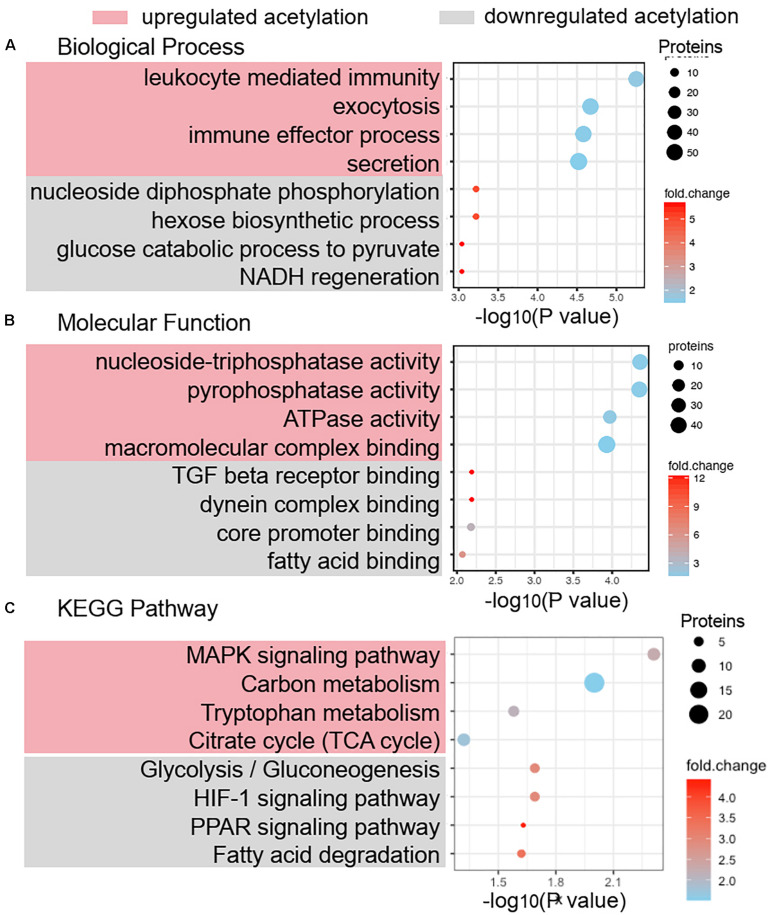
Functional annotation and enrichment analysis of differentially acetylated proteins. Biological process analysis **(A)** and molecular function analysis **(B)** of differentially acetylated proteins in PCOS and control groups were enriched by GO term. **(C)** KEGG pathway enrichment of proteins with significant change of lysine acetylation.

**FIGURE 4:**
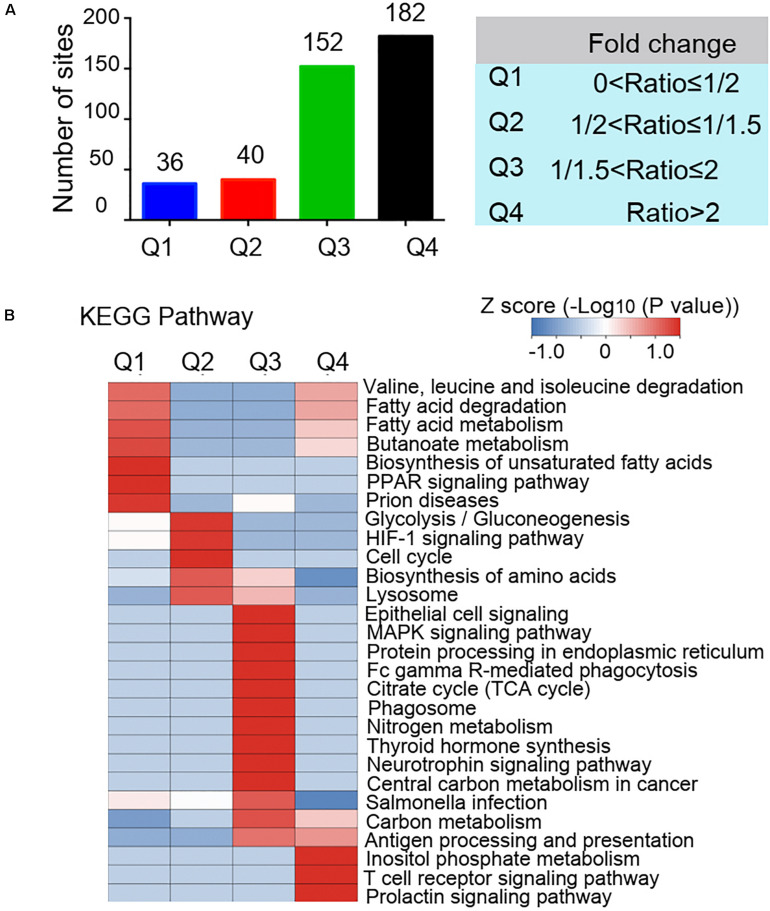
Quantitative classification of differentially acetylated proteins. The differentially modified proteins were divided into four quantified parts according to their differential expression multiples: Q1 (0 < ratio ≤ 1/2), Q2 (1/2 < ratio ≤ 1/1.5), Q3 (1.5 < ratio ≤ 2), and Q4 (ratio > 2). **(A)** Numbers of acetylated proteins in each quantified part (Q). **(B)** Clustering analysis base on KEGG pathway enrichment of quantitative differentially acetylated proteins in each Q group.

The GO functional classification showed the enrichment trends of the differentially acetylated proteins in the biological process and molecular function categories. The proteins with significant changes in acetylation level in the PCOS group were mainly involved in the metabolic processes and immune regulation (Figures 3A,B). We further performed a function-enrichment analysis based on the KEGG database. Most notably, differentially acetylated proteins were significantly enriched in the metabolic pathways such as carbon metabolism, tryptophan metabolism, glycolysis/gluconeogenesis, and fatty acid degradation, and molecular signaling pathways regulated glucose and lipid metabolism such as HIF-1 and PPAR signaling pathways (Figure 3C and Supplementary Table 2). To further confirm the correlations of protein functions with the alteration level of acetylation modification, we divided the differentially modified proteins into four quantified parts according to their differential expression multiples: Q1 (0 < ratio ≤ 1/2), Q2 (1/2 < ratio ≤ 1/1.5), Q3 (1.5 < ratio ≤ 2), and Q4 (ratio > 2) (Figure 4A). We then conducted KEGG enrichment analyses for each Q group and performed a cluster analysis (Figure 4B). The enrichment analyses showed that the differentially acetylated proteins were mainly enriched in the branched-chain amino acid degradation and fatty acid degradation/metabolism in the Q1 group, in the glycolysis/gluconeogenesis in the Q2 group, in the TCA cycle and carbon metabolism in the Q3 group, and inositol phosphate metabolism in the Q4 group.

### Lysine Acetylation of Proteins Regulates Metabolic Pathways in PCOS Granulosa Cells

**FIGURE 5:**
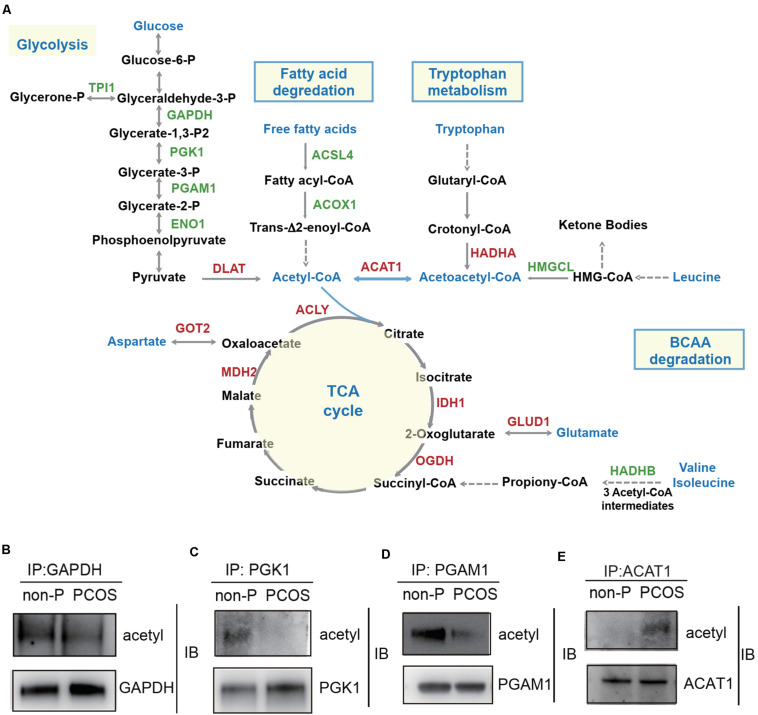
Lysine acetylation of proteins regulates metabolic pathways in PCOS granulosa cells. **(A)** The diagram of metabolism pathways with differentially acetylated enzymes highlighted. Red color represented the enzyme with hyperacetylation in the PCOS group; green color represented the enzyme with hypoacetylation in PCOS group; and blue color represented the important metabolites and metabolic pathways. **(B–E)** Verification of lysine acetylation changes of key enzymes. Immunoprecipitated proteins and acetylation status of target proteins were detected by Western blot. GAPDH (glyceraldehyde-3-phosphate dehydrogenase), PGK1 (phosphoglycerate kinase 1), PGAM1 (phosphoglycerate mutase 1), ACAT1 (acetyl-CoA acetyltransferase 1).

Since several metabolic pathways exhibited acetylation changes by KEGG analysis, we narrowed our investigation to the subset of enzymes involved in glycolysis, fatty acid degradation, TCA cycle, and amino acid metabolism, to examine whether lysine acetylation level of key enzymes influenced the metabolism homeostasis of granulosa cells. The enzymes with significant changes of acetylation level in the PCOS group were highlighted in a metabolic pathway diagram (Figure 5A). In glycolysis, five enzymes involved in the reactions for the conversion of glucose-6-phosphate to pyruvate were hypoacetylated in PCOS granulosa cells, including glyceraldehyde-3-phosphate dehydrogenase (GAPDH), phosphoglycerate kinase 1 (PGK1), phosphoglycerate mutase 1 (PGAM1), triosephosphate isomerase 1 (TPI1), and alpha-enolase 1 (ENO1). The dihydrolipoyllysine-residue acetyltransferase component of pyruvate dehydrogenase complex (DLAT), which catalyzed the conversion of pyruvate to acetyl-CoA, was hyperacetylated in the PCOS group. Two enzymes that catalyzed the fatty acid degradation and produced acyl-CoA, long-chain fatty acid CoA ligase 4 (ACSL4) and peroxisomal acyl-CoA oxidase1 (ACOX1), were hypoacetylated in the PCOS group. Besides, the acetylation levels of several key enzymes in TCA cycle and amino acid metabolism (tryptophan metabolism, branched-chain amino acid degradation, and aspartate and glutamate metabolism) were significantly changed in the PCOS group. Acetyl-CoA linked the glycolysis, fatty acid degradation, and amino acid metabolism to the TCA cycle, and these metabolic pathways had in common a single enzyme, acetyl-CoA acetyltransferase 1 (ACAT1). ACAT1 catalyzed a reversible reaction of the cleavage of acetoacetyl-CoA into two acetyl-CoAs. The lysine acetylation level of ACAT1 was notably increased at the specific site of Lys-174 in the PCOS group. In addition, co-immunoprecipitation and Western blotting analysis further verified the changes in protein acetylation levels of four enzymes in PCOS ovarian granulosa cells compared to control cells, ACAT1, GAPDH, PGK1, and PGAM1 (Figure 5B).

### Correlation of ACAT1 Acetylation Levels in PCOS Granulosa Cells With Clinical Outcomes

**FIGURE 6:**
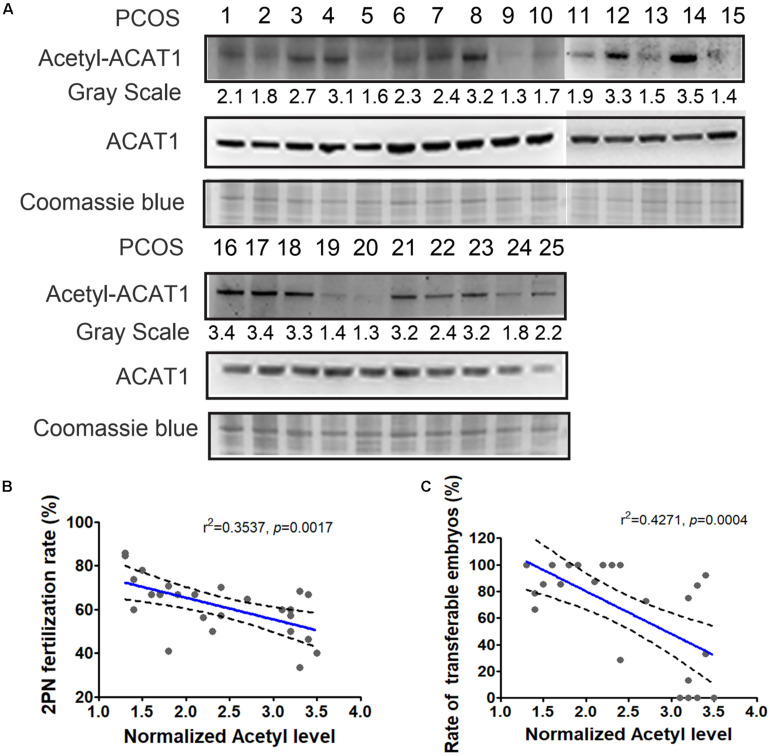
Correlation of ACAT1 acetylation levels in PCOS granulosa cells with clinical outcomes. **(A)** Detection of ACAT1 acetylation in granulosa cells of 25 PCOS patients. Loading control was Coomassie blue staining. The acetylation value (grayscale ratio) was normalized with Coomassie blue staining. When the ratio of the acetylation level of ACAT1 Lys-174 to the expression level of ACAT1 protein was more than 2, it was classified as hyperacetylation subgroup, while the hypoacetylation subgroup was defined when the ratio was less than 2. **(B,C)** showed the correlation analyses of ACAT1 acetylation levels with the 2PN fertilization rate and rate of transferable embryos. R: Pearson correlation coefficient.

The changes in the lysine acetylation of enzymes might affect their activities and regulated metabolic reactions in PCOS. Metabolic disorders of granulosa cells interfere with the metabolic homeostasis in the microenvironment of follicles, thereby affecting oocyte maturation and development potential. Since ACAT1 was an enzyme central to metabolic pathways, we further analyzed whether the changes of ACAT1 acetylation influence the process of oocyte maturation and embryo development. We collected the granulosa cell samples of 25 PCOS patients, and examined the ACAT1 acetylation level of each sample (Figure 6A). The acetylation level of ACAT1 Lys-174 was normalized with immunoprecipitated ACAT1 protein level by ImageJ. When the ratio of the acetylation level of ACAT1 Lys-174 to the expression level of ACAT1 protein was more than 2, it was classified as a hyperacetylation subgroup, while the hypoacetylation subgroup was defined when the ratio was less than 2. The clinical outcomes of these two subgroups were further analyzed. As shown in Table 2, the PCOS women with enhanced ACAT1 acetylation in their granulosa cells had a markedly reduced 2PN fertilization rate (56.5% vs. 69.3%, respectively), cleavage rate (56.2% vs. 69.3%, respectively), and rate of transferable embryos (52.5% vs. 91.7%, respectively). At the same time, there was a significantly negative correlation of ACAT1 acetylation levels in PCOS granulosa cells with 2PN fertilization rate and rate of transferable embryos (Figures 6B,C), indicating that increased ACAT1 acetylation in granulosa cells affected the ovarian metabolic microenvironment and inhibited oocyte quality and embryo development efficiency.

**TABLE 2:** The clinical outcomes of PCOS women with different acetylation level of ACAT1.

	**Hyperacetylation**	**Hypoacetylation**	***p*-value**
Number	15	10	
No. of MII oocyte	13.6 ± 9.1	13.1 ± 6.9	0.88
No. of 2PN embryos	7.9 ± 5.7	8.9 ± 4.5	0.66
2PN fertilization rate	56.5%	69.3%	**0.01**
No. of 2PN cleavage embryos	7.9 ± 5.4	8.9 ± 4.3	0.63
Cleavage rate	56.2%	69.3%	**0.01**
No. of transferable embryos	4.7 ± 5.1	8.1 ± 4.2	0.09
Rate of transferable embryos	52.5%	91.7%	**0.01**
Clinical pregnancy rate	12.5% (2/15)	50% (5/10)	0.17

*The granulosa cell samples of 25 PCOS patients were examined to analyze the correlation of the lysine acetylation of ACAT1 protein to the clinical outcomes. The acetylation value (grayscale ratio) was normalized with loading control, Coomassie blue staining. When the ratio of the acetylation level of ACAT1 Lys-174 to the expression level of ACAT1 protein was more than 2, it was classified as hyperacetylation subgroup, while the hypoacetylation subgroup was defined when the ratio was less than 2. The IVF outcome measure: 2PN fertilization rate was defined as the number of two pronuclear (2PN) embryos divided by the number of MII oocytes; cleavage rate was defined as the number of 2PN cleavage embryos divided by the number of MII oocytes; transferable embryos were defined as day-3 embryos that reached the 4-cell stage, and the rate of transferable embryos was defined as the number of transferable embryos divided by the number of 2PN embryos; clinical pregnancy was defined as a presence of intrauterine gestational sac observed on ultrasound after 30 days of embryo transfer. The bold *p*-value means the significant difference between two groups.*

## Discussion

PCOS is a complex heterogeneous disease that is associated with reproductive disorders and metabolic abnormalities. Local metabolic abnormalities in the ovary affect ovarian function, including follicle development, oocyte maturation, and ovulation. Our previous studies also showed abnormal levels of intermediate metabolites in the follicular fluid from women with PCOS, involved in the metabolic pathways of glycolysis, fatty acid oxidation, BCAA catabolism, and TCA cycle (Zhao H. et al., 2015). It is needed to further clarify the mechanism of ovary local metabolic disorders in PCOS. Identifying posttranslational modifications is critical to better understanding the PCOS etiology and providing new targets for disease treatment and prevention. Protein lysine acetylation is an evolutionarily, highly conserved posttranslational modification mechanism that regulates protein function and integrates metabolic flux. Increasing evidence suggested that most enzymes involved in glucose and fatty acid metabolism were acetylated at critical lysine sites, which played significant roles in controlling energy metabolism (Wang et al., 2010; Choudhary et al., 2014; Carrico et al., 2018). However, there are still no reports on the roles of protein acetylation in PCOS. In the current study, we performed a quantitative analysis of acetylated proteomics in PCOS ovarian granulosa cells to explore the pathogenesis on the posttranslational level. Our results detected differentially acetylated proteins notably enriched in several metabolic pathways and suggested that acetylation modification indeed played an important role in the regulation of metabolic abnormalities in PCOS, which is a novel finding and may provide new insights into the mechanistic basis of PCOS.

Our analyses highlighted a subset of enzymes with significant changes of acetylation, including a mitochondrial acetyl-CoA acetyltransferase ACAT1. Active ACAT1 is a tetrameric enzyme that converts two acetyl-CoA molecules to acetoacetyl-CoA and CoA reversibly, and tetramer formation of ACAT1 from monomers increased its activity (Haapalainen et al., 2007; Fan et al., 2016). Previous report demonstrated that acetylation on several lysine sites in Acat1 decreased its activity in mice, and molecular modeling revealed that the inhibitory effects of acetylation on Acat1 activity were due to decreased affinity for CoA (Still et al., 2013). In our study, we found that the acetylation on Lys-174 of ACAT1 increased over 5-fold in the granulosa cells of PCOS women. Based on the crystal structure resolved at 1.85 Å of human ACAT1 (PDB: 2IB8), Lys-174 was located in the binding region of monomers forming tetramer (Supplementary Figure 4), suggesting that the hyperacetylation on Lys-174 might prevent the formation of tetrameric ACAT1 and decrease its enzyme activity. Considering that ACAT1 is at the cross-road of glycolysis, fatty acid degradation, tryptophan metabolism, BCAA degradation, and TCA cycle, alteration of ACAT1 activity in the granulosa cells could influence the metabolic pathways mentioned above and disturb the metabolic homeostasis in the ovarian follicles.

The oocytes in PCOS patients are often of poor quality, leading to lower fertilization, cleavage, and implantation rates, though an increased number of oocytes is retrieved during the *in vitro* fertilization and the alterations in oocyte competence are considered potential causative factors for infertility in women PCOS (Qiao and Feng, 2011; Palomba et al., 2017). In our previous study, we have detected *ACAT1* as a key gene that regulated the oocyte maturation and maintained the developmental potential of the oocyte (Zhao et al., 2019). The deficient expression of *ACAT1* in the oocyte blocked the production of acetyl-CoA and succinate and therefore weakened the energy metabolism of the oocyte. Moreover, in our present study, we found that the acetylation level of ACAT1 in PCOS granulosa cells was negatively associated with oocyte quality and embryo development, indicating that ACAT1 acetylation affected the microenvironment of oocyte maturation by reducing the enzyme activity and inducing the metabolic disorders. Collectively, our studies revealed ACAT1 as an important metabolic regulator functioned by transcriptional regulation and posttranslational modification, and it will be a crucial target for future investigations into the pathogenesis of PCOS.

Lysine acetyltransferases and lysine deacetylases are responsible for reversible changes in protein acetylation status. Lysine acetyltransferase can be grouped into three major families such as the general control of amino acid synthesis 5 (GCN5), the p300/CREB-binding proteins (CBP/p300), and the MYST subfamily, while lysine deacetylases mainly belong to two distinct families such as Zn^2+^-dependent histone deacetylases (HDAC) and NAD^+^-dependent sirtuin deacetylases (Chang and Guarente, 2014; Choudhary et al., 2014; Mattioli et al., 2019). Our results of KEGG pathway enrichment showed that the hypoacetylation of five enzymes channeled glycolysis flux bidirectionally in PCOS granulosa cells, including GAPDH, PGK1, PGAM1, TPI1, and ENO1 (Figure 5). The reduced GAPDH acetylation was also observed in the liver of obesity and type 2 diabetes mouse models, and key lysine residues in GAPDH contribute to the regulation of glucose tolerance and sensitivity to glucagon and insulin (Bond et al., 2017). In addition, acetyltransferase P300 regulated cellular glucose metabolism by mediating enzyme acetylation. P300 regulated the acetylation of PGK1 and promoted its enzymatic activity and cell metabolism in liver cancer cells (Hu et al., 2017). *P300* knockout in HEK293T cells reduced ENO1 activity and decreased glycolysis and increased the sensitivity to glucose depletion-induced cell death (Huang et al., 2018). SIRT1 could directly modulate PGAM1 deacetylation and attenuate its activity, thus restricting flux through glycolysis (Hallows et al., 2012). Consistent with these findings, the decreased acetylation of GAPDH, PGK1, PGAM1, and ENO1 detected in PCOS granulosa cells indicated the reduced catalytic activity of these enzymes and impaired glycolysis. Moreover, we comprehensively analyzed the changes in intermediate metabolite levels in the follicular fluid of PCOS patients based on previous studies and clarified the abnormality in glycolysis (Zhao H. et al., 2015). Further researches are required to determine the mechanism of differential acetylated sites of these enzymes in regulating cellular glucose metabolism in PCOS.

In addition, our results showed that two rate-limiting enzymes which catalyzed the fatty acid degradation were hypoacetylated and four enzymes involved in TCA cycle were hyperacetylated in PCOS granulosa cells (Figure 5A). The acetylation alteration of these enzymes might impair the fatty acid oxidation and energy metabolism in mitochondria. Moreover, the proteins with lower acetylation level were enriched in hypoxia-inducible factor 1 (HIF-1) and peroxisome proliferator-activated receptor (PPAR) signaling pathways (Figure 3C). HIF-1 and PPAR signaling pathways have pleiotropic functions in mediating glucose and lipid metabolism, inflammation, and oxidative stress (Gross et al., 2017; Chen et al., 2018; Al et al., 2019). We have proved the mitochondrial dysfunction, upregulation of pro-inflammatory factors, and higher level of reactive oxygen species (ROS) in the granulosa cells of PCOS women (Zhao H. et al., 2015; Zhao Y. et al., 2015), suggesting that the lysine acetylation of key proteins may have a vital function in modulating the imbalance of energy metabolism, inflammation, and oxidative stress microenvironment in the follicles of PCOS.

In conclusion, protein lysine acetylation plays an important role in the metabolic regulation of PCOS. Changes in acetylation modification of key enzymes in granulosa cells might affect their activities and alter follicular metabolic homeostasis, thereby affecting oocyte quality and embryo development in PCOS. However, further exploration of the molecular mechanism of the lysine acetylation modification in regulating ovarian energy metabolism and follicular development is needed and would provide a new perspective and important mechanism for elucidating the pathogenesis of PCOS.

## Data Availability Statement

The datasets presented in this study can be found at http://msviewer.ucsf.edu/prospector/cgi-bin/msform.cgi?form= msviewer, MS-Viewer (the search key for the saved data set is q40sd3k4bg).

## Ethics Statement

The studies involving human participants were reviewed and approved by Peking University Third Hospital Reproductive Medicine Ethics Committee. The patients/participants provided their written informed consent to participate in this study.

## Author Contributions

ZM performed the bioinformatics analyses and molecular experiments. XL and HZ collected the granulosa cell samples and the clinical information. XZ and RL recruited the subjects. YZ and ZM wrote the manuscript. YY, YZ, and YF designed the study and revised the manuscript. JQ and ML contributed to experimental guidance and discussion. All authors contributed to the article and approved the submitted version.

## Conflict of Interest

The authors declare that the research was conducted in the absence of any commercial or financial relationships that could be construed as a potential conflict of interest.
