# Supplementary material for: Protein Lysine Acetylation in Ovarian Granulosa Cells Affects Metabolic Homeostasis and Clinical Presentations of Women With Polycystic Ovary Syndrome
Source: Front Cell Dev Biol. 2020 Sep 11;8:567028. doi: 10.3389/fcell.2020.567028 (PMC7518144; doi:10.3389/fcell.2020.567028)

Supplementary Tables

Supplementary Table 1. Differential proteins and acetylated sites information of PCOS and non-PCOS patients.

Supplementary Table 2. Protein information of GO enrichment and KEGG pathway.

Supplementary Table 3. Normalized acetylation level and clinical results of PCOS patients.

Supplementary Figures

Supplementary Figure 1. A scater diagram of repeated test in comparing protein quantitative value between two group. P1-P3 and C1-C3 were triplicate experiment group. R: Pearson correlation coefficient.

Supplementary Figure 2. Quantitative proteomic analysis on GCs samples. A) Identified modification sites in peptides. B) QC validation of MS data. Mass error indicated distribution of peptides. C) Identified Peptides length distribution.

Supplementary Figure 3. Motif analysis of identified acetylated peptides. A) Consensus sequence logos plot for acetylation sites ± ten amino acids from identified lysine residues upregulated acetylated proteins in PCOS/non-PCOS GCs. B) Logos plot from identified lysine residues downregulated acetylated proteins. C) Logos plot of the lysine of all KAc identified sites. D) Heatmap of amino acids frequence of the sequences flanking KAc sites. E) Sequence logos of acetylated motifs.

Supplementary Figure 4. The crystal structure resolved at 1.85 Å of human ACAT1 (PDB: 2IB8). Lys-174 was located in the binding region of ACAT1 monomers forming tetramer.

Supplementary Figure 1

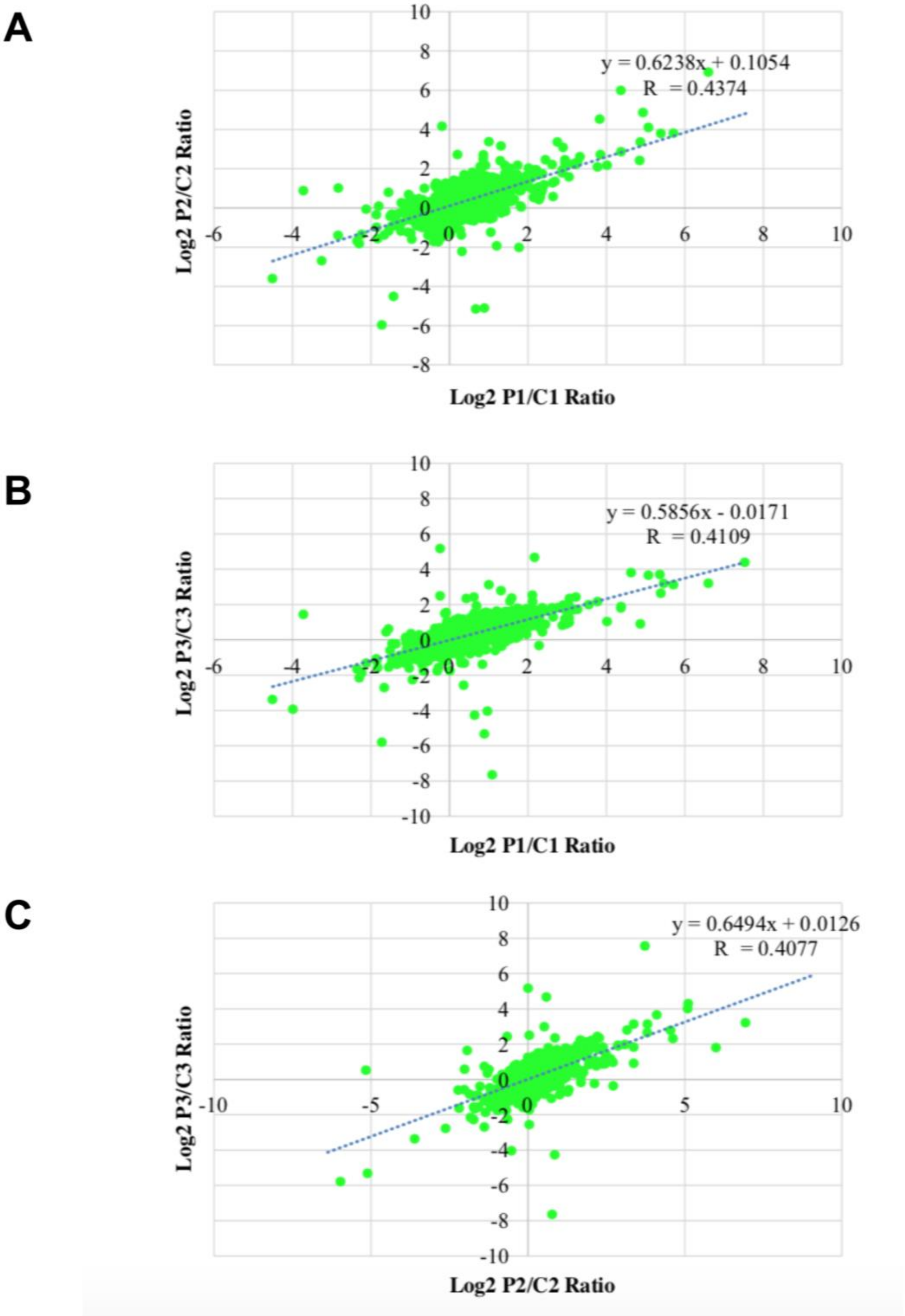

Supplementary Figure 2

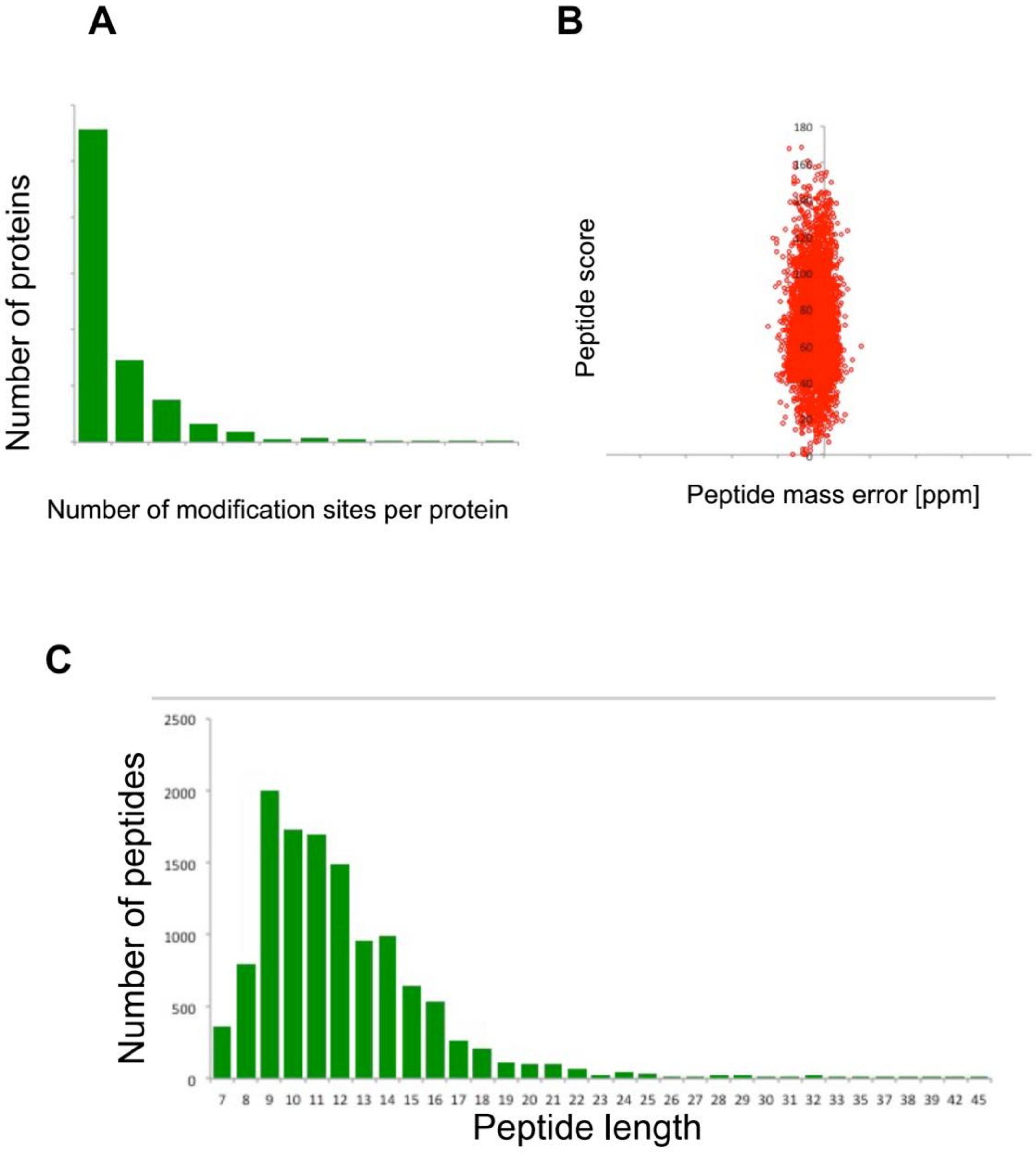

Supplementary Figure 3

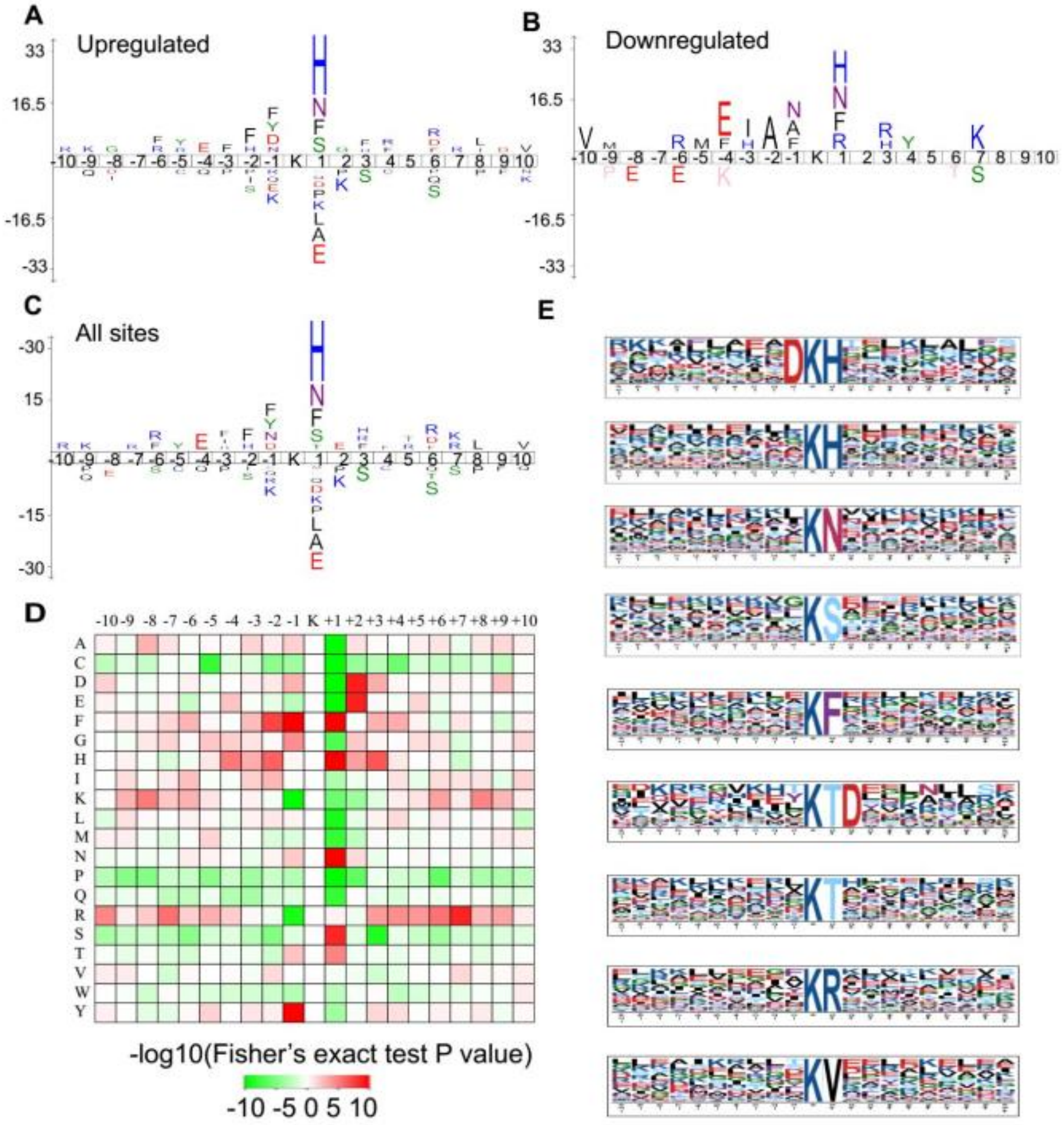

Supplementary Figure 4

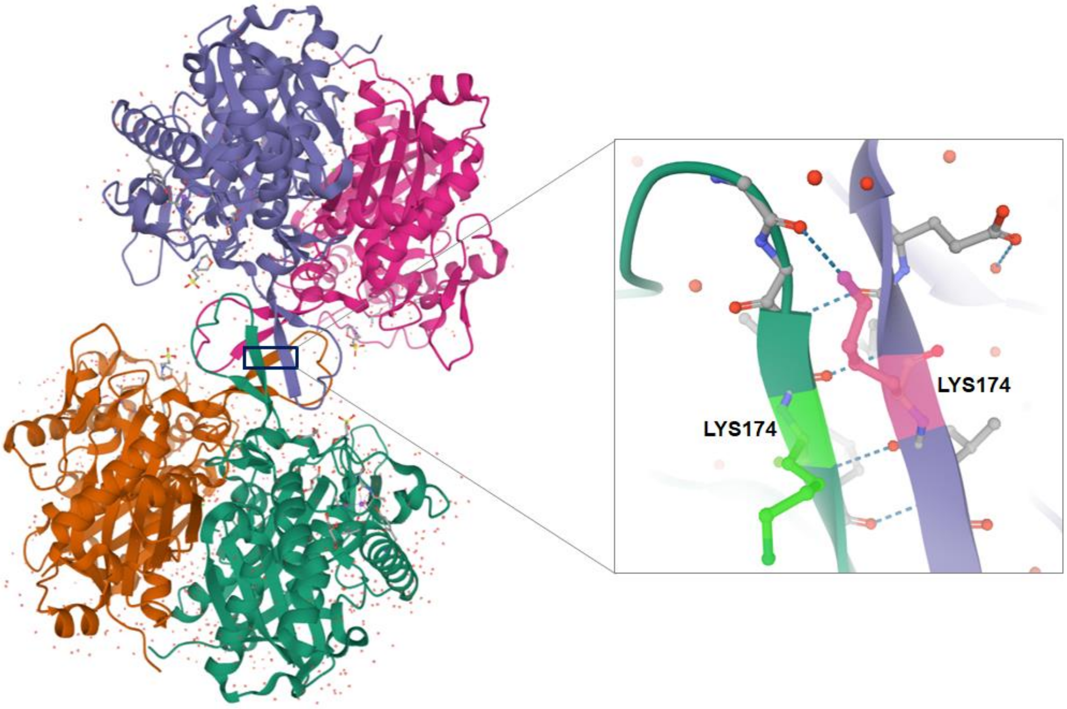

Supplement: Supplementary file 1 [file Data_Sheet_1.PDF]
